# Miscellany

**Published:** 1905-01

**Authors:** 


### Miscellany.

An Interesting Fact in Hayden's Life.—We find an entertaining story about a once widely known book in the *Cincinnati Enquirer.* Two great-granddaughters of Dr. Horace H. Hayden, it seems, discovered in the Mercantile Library of St. Louis a copy of their great-grandfather's "Geological Essays," for which they offered to pay $500. The offer was declined, as the books in the library are not for sale. We fancy that by judiciously using the weekly Book Exchange column of the *New York Times Book Review* Dr. Hayden's granddaughters might be able to secure a copy for a much smaller price. "Geological Essays: An Inquiry into Geological Phenomena to be Found in Various Parts of America," was published in Baltimore in 1829. There may be many copies extant. Horace Hayden was born in Windsor, Conn., in 1769, and died in Baltimore in 1844. He was a school-teacher and an architect before he took up the study of dentistry after his twenty-first year. He became a successful and famous dentist. He studied medicine, too, and served as an army surgeon in the War of 1812. His favorite avocation throughout his life was the study of geology. Though he wrote books on dentistry and other subjects, his fame as an author is chiefly associated with his "Geological Essays," which Benjamin Silliman said "should be used as a text-book in all our schools."—*Exchange.*

